# A Unique Presentation of Diffuse Intraductal Papillary Mucinous Neoplasm of Bile Duct Successfully Treated With Orthotopic Liver Transplant and Pancreaticoduodenectomy Procedure: A Case Report

**DOI:** 10.7759/cureus.70177

**Published:** 2024-09-25

**Authors:** Yichen Fang, Catherine O'Leary, Hannah Lowe, Danielle H Carpenter, Henry B Randall, Mustafa D Nazzal

**Affiliations:** 1 Department of Surgery, Saint Louis University School of Medicine, Saint Louis, USA; 2 Department of Medicine, Saint Louis University School of Medicine, Saint Louis, USA; 3 Department of Surgery, Saint Louis University Hospital, Saint Louis, USA; 4 Department of Pathology, Saint Louis University Hospital, Saint Louis, USA

**Keywords:** bile duct, cholangiocarcinoma, dysplasia, intraductal papillary mucinous neoplasm of the bile duct (ipmn-b), orthotopic liver transplant (olt), pancreaticoduodenectomy

## Abstract

Biliary intraepithelial neoplasm (BilIN) and intraductal papillary mucinous neoplasm of the bile duct (IPMN-B) are recognized as the two main precursors to biliary carcinoma. Surgical resection is the mainstay of treatment, with pancreaticoduodenectomy for extrahepatic biliary duct disease involving the pancreatic or distal portion of the bile duct, and bile duct and liver resection for perihilar and intrahepatic bile duct involvement. For diffuse IPMN-B with involvement of the entirety of the biliary epithelium of both intrahepatic and extrahepatic biliary systems, there is no well-documented consensus on treatment due to its rarity. Therefore, we present a case of a 56-year-old male with diffuse IPMN-B managed with combined orthotopic liver transplant and pancreaticoduodenectomy. As such, the clinical presentation, diagnostics, and unique course of intervention for our patient are described. This case provides insight into the topic of managing IPMN-Bs, particularly with diffuse biliary tree involvement. With such a rare disease with wildly varied presentations, consensus on a set treatment algorithm is nearly impossible to establish. This case describes one treatment pathway we found to be successful.

## Introduction

Biliary intraepithelial neoplasm (BilIN) and intraductal papillary mucinous neoplasm of the bile duct (IPMN-B) are recognized as the two main precursors to biliary carcinoma. Biliary disease is categorized as either mucin-hypersecreting type or non-mucin producing type. IPMNB is a biliary counterpart to mucin hypersecreting biliary papillomatosis (BP) [1]. IPMN-B are non-invasive epithelial neoplasms characterized by papillary projections within the bile [2]. Intraductal papillary neoplasms of the bile duct account for 7-11% of bile duct tumors in Western countries. Risk factors for IPMN-B include hepatolithiasis, clonorchiasis infection, primary sclerosing cholangitis, and familial adenomatous polyposis [3]. The median age at presentation in most cases is 60-66 years, with a male predominance. Symptoms include recurrent and intermittent abdominal pain, specifically in the right upper quadrant, and jaundice. However, most IPMN-Bs can be asymptomatic and found incidentally. One of the most common IPMN-Bs is a mass associated with ductal dilation proximal and distal to the mass [3]. Laboratory values will reflect biliary obstruction. IPMN-Bs will present initially on abdominal or endoscopic ultrasound as echogenic intraductal masses and/or bile duct dilations [1]. IPMN-B, although rare, is a recognized precursor lesion for cholangiocarcinoma (CC), an invasive malignancy of epithelial cells within the biliary tree [4]. Invasive carcinomas are found in around 40-80% of IPMN-Bs. Pancreato-biliary type is the most common IPMN-B found in the western countries [1]. Also, IPMN-B poses an increased risk of recurrent biliary obstruction and associated complications such as cholangitis. The first-line treatment for IPMN-Bs without distant spread includes surgical resection with negative margins [3]. Tumor location and extent are the key factors when determining the surgical procedure plan. Multimodality approaches including MRI with magnetic resonance cholangiopancreatography (MRCP), endoscopic retrograde cholangiopancreatography (ERCP), cholangioscopy, and whole-body PET/CT should aid in this decision [3]. One study showed a five-year survival rate of 81% post-resection of IPMN-B [1]. IPMN-B typically predisposes patients to hilar and periampullary CC. Tissue biopsies are used as a final confirmatory diagnosis [4]. Surgical treatment for IPMN-Bs follows similar rules applicable to CC. Surgical resection is the mainstay of treatment with pancreaticoduodenectomy for extrahepatic biliary duct disease involving the pancreatic or distal portion of the bile duct, and bile duct and liver resection for perihilar and intrahepatic bile duct involvement. For diffuse IPMN-B with involvement of the entirety of the biliary epithelium of both intrahepatic and extrahepatic biliary systems, without previously diagnosed CC, there is no well-documented consensus on treatment due to its rarity. Additionally, there is little evidence of combined orthotopic liver transplant (OLT) and pancreaticoduodenectomy for treatment. Therefore, we present a case of diffuse IPMN-B managed with combined OLT and pancreaticoduodenectomy. As such, the clinical presentation, diagnostics, and unique course of intervention for our patient will be described.

## Case presentation

This is a case of a 56-year-old male with no prior medical history and no tobacco or heavy alcohol use who initially presented for sporadic, infrequent postprandial bloating and light-colored stools. Labs were significant for an elevated total bilirubin, alkaline phosphatase, and liver enzymes. An abdominal ultrasound showed common bile duct (CBD) dilation. Subsequent endoscopic ultrasound showed bile duct obstruction from an intraductal soft tissue lesion, which was concerning for malignancy.

**Figure 1:**
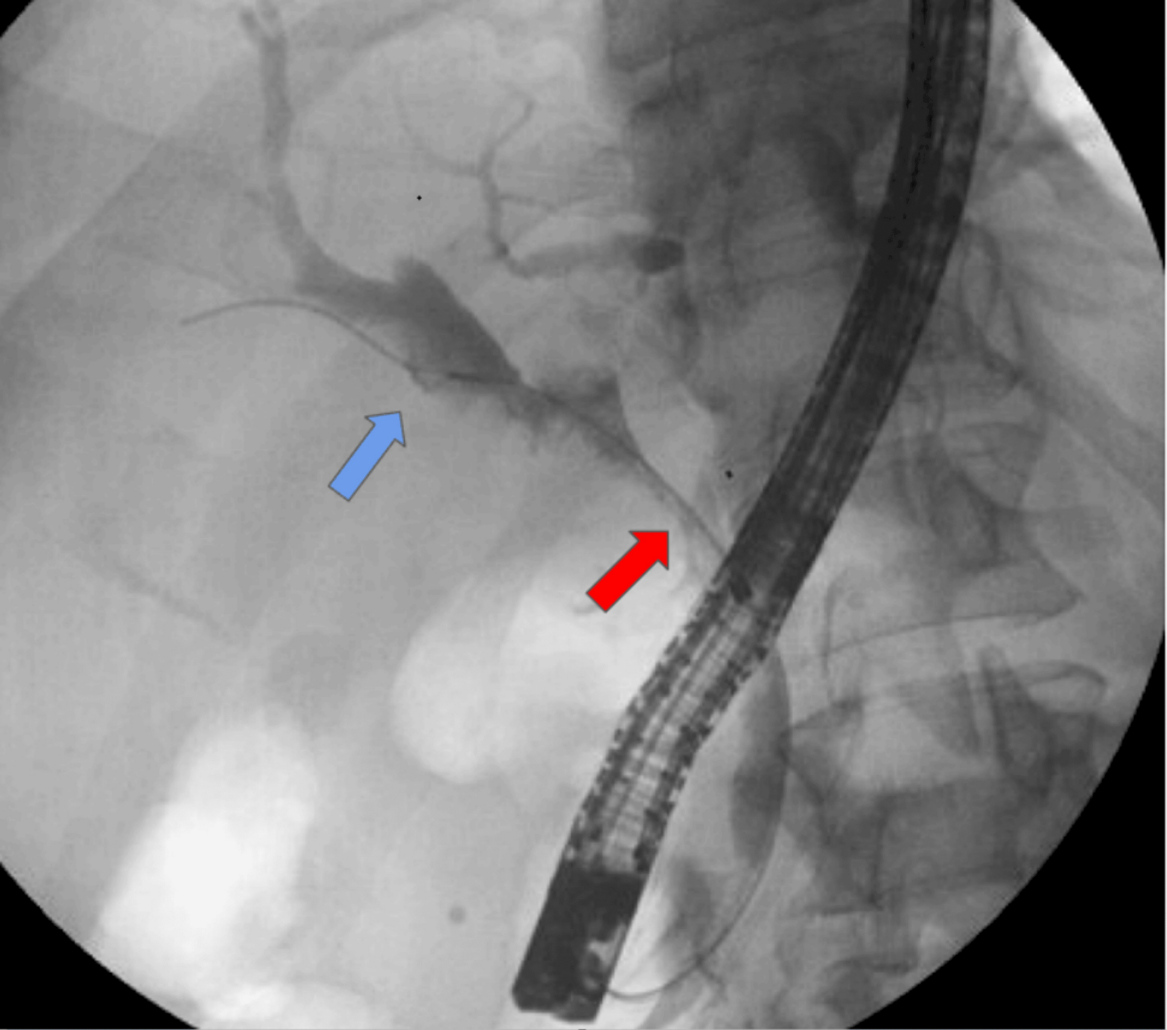
ERCP showing filling defect of proximal CBD (red arrow) with dilation of the left and right hepatic ducts (blue arrow) ERCP, endoscopic retrograde cholangiopancreatography; CBD, common bile duct

**Figure 2:**
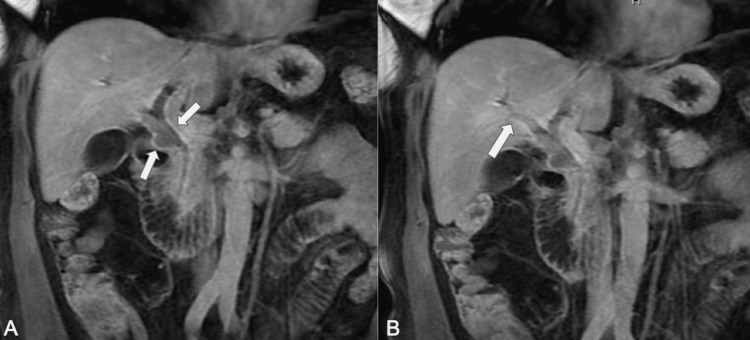
MRCP showing enhancing soft tissue in the common bile duct (A) extending into the right intrahepatic duct (B) MRCP, magnetic resonance cholangiopancreatography

**Figure 3:**
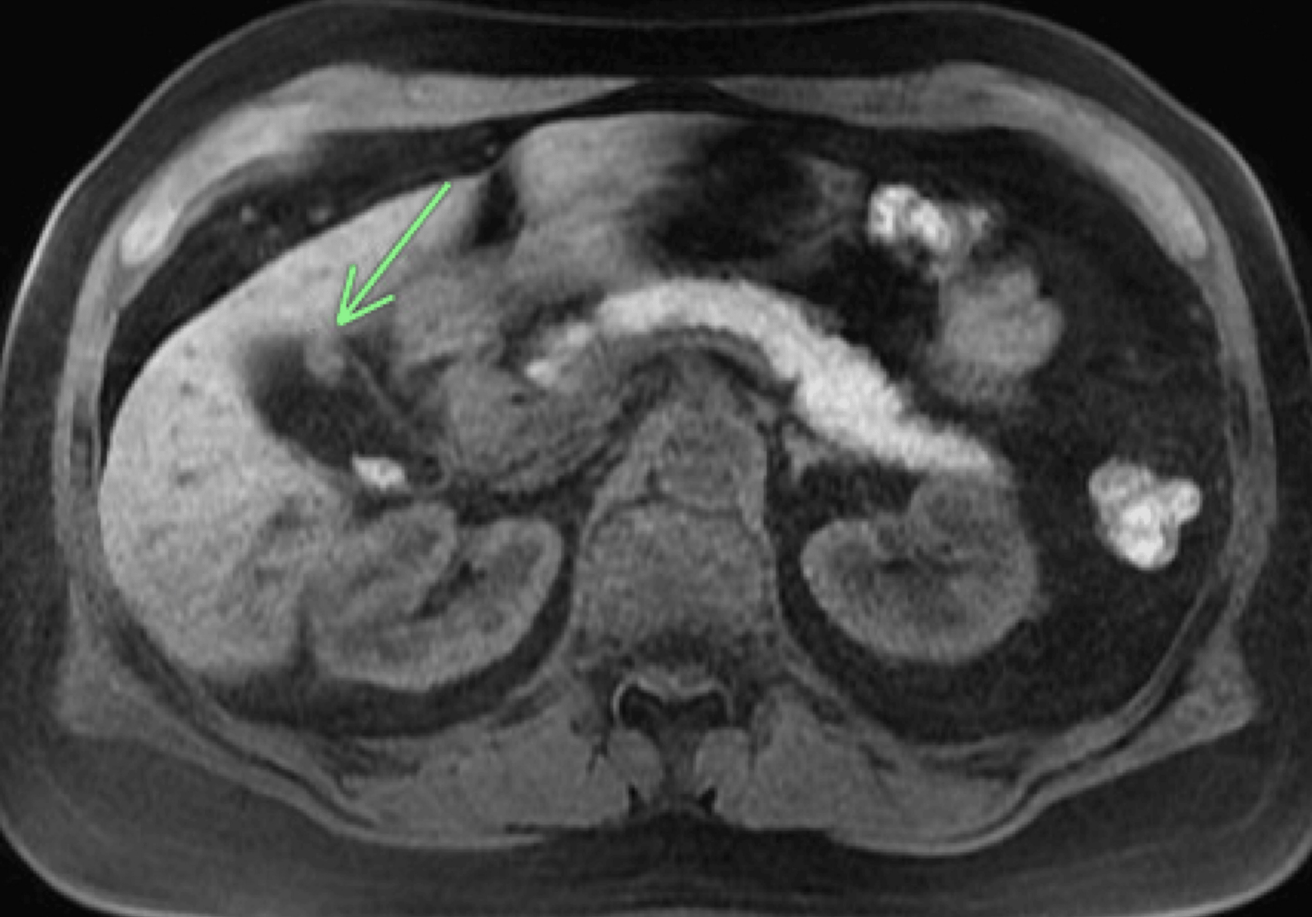
MRCP showing enhancing mass within the gallbladder MRCP, magnetic resonance cholangiopancreatography

**Figure 4:**
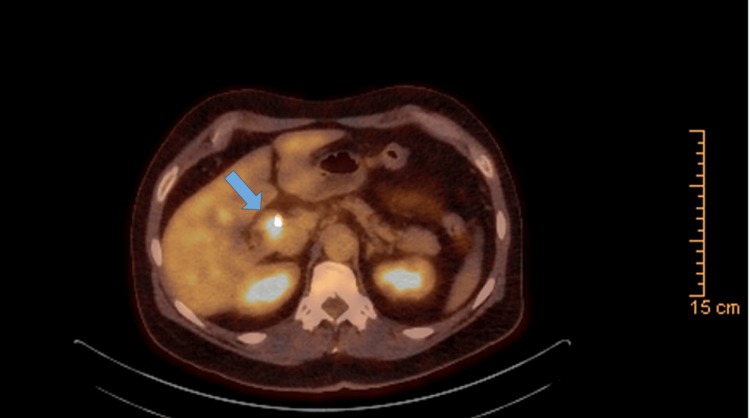
PET-CT shows intense FDG uptake along biliary stent (blue arrow). Biopsy of CBD stricture showing IPMN in a similar location. The FDG avid lesion within the gallbladder was suspicious for malignancy. PET-CT, positron emission tomography-computed tomography; FDG, fluorodeoxyglucose; CBD, common bile duct; IPMN, intraductal papillary mucinous neoplasm

The patient underwent evaluation with ERCP with SpyGlass, which showed a proximal CBD filling defect and dilation of the left and right hepatic ducts (Figure 1) requiring sphincterotomy and stent placement. Histologic findings from the tissue biopsies were consistent with low-grade intraductal papillary neoplasm. A subsequent MRCP showed enhancing soft tissue in the CBD extending into the right intrahepatic duct (Figure 2) with additional masses in the gallbladder and liver (Figure 3). Liver lesion biopsy showed focal bridging necrosis and nodularity with no evidence of malignancy. Repeat ERCP with SpyGlass showed a single diffuse biliary stricture in the hepatic duct system involving both the left and right systems (Bismuth IV), extending from distal CBD up to common hepatic duct, across the bifurcation and into the left and right main hepatic ducts. Histopathologic findings were again consistent with low-grade intraductal papillary neoplasm. Cancer antigen 19-9 (CA 19-9) was 29 U/mL (normal 0-37 U/mL). Biliary fluorescence in situ hybridization (FISH) analysis of the bile duct brushings was negative for chromosomal alterations typically associated with carcinoma, and PET scan was significant for intense fluorodeoxyglucose (FDG) uptake along the bile duct and gallbladder (Figure 4). As such, a diagnostic laparoscopy and cholecystectomy were performed to rule out CC.

**Figure 5:**
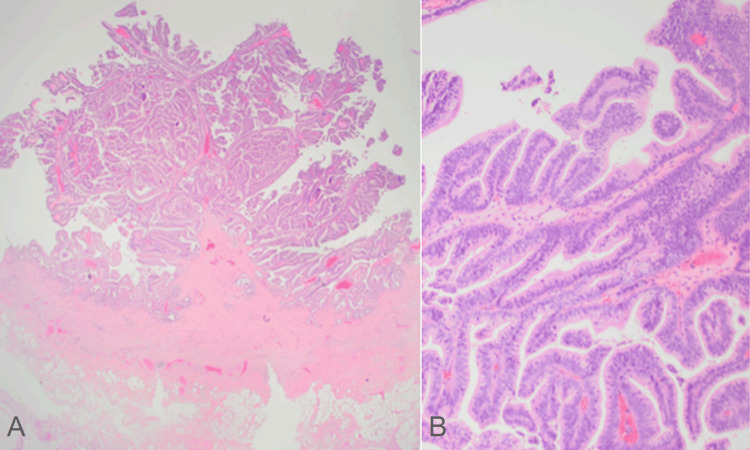
Low magnification image (A) of the gallbladder ICPN, with no invasive growth (H&E, 12.5x). Higher magnification image (B) of the ICPN showing papillary architecture and only low-grade dysplasia (H&E, 100x). ICPN, intra-cholecystic papillary neoplasm

The cytology of the bile duct showed papillary mucinous epithelium with low-to-moderate atypia but no high-grade dysplasia. Pathology from an intra-cholecystic papillary neoplasm with low-grade dysplasia (1.7 cm) showed no invasive carcinoma (Figure 5). In addition, there was a separate focus of low-grade (flat) biliary intraepithelial neoplasia present at the cystic duct margin, though again, without invasive carcinoma or high-grade dysplasia.

In the absence of malignancy and concern for potential malignant transformation with involvement of the entirety of the biliary system, and in the presence of biliary obstruction requiring repeated biliary stenting, the decision was made for a staged procedure with OLT followed by pancreaticoduodenectomy to eliminate the diseased biliary system completely. Once the patient was listed, application for Model for End-Stage Liver Disease (MELD) exception points was requested and granted. Continued surveillance with ERCP and biopsy remained consistent with intraductal papillary neoplasm of the bile duct without evidence of CC. Once a liver became available, a staged OLT using a piggyback technique with choledocho-choledochostomy was performed followed by a pancreaticoduodenectomy six days later as planned. The explant pathology demonstrated a focus of invasive carcinoma of the bile duct consistent with an early-stage CC.

The postoperative course was complicated by pancreatic and biliary leak, for which a percutaneous transhepatic cholangiogram catheter (PTC) with an external/internal biliary drain was inserted to completely drain the biliary system. An extrahepatic drain was placed directly in the fluid collection. The pancreatic leak was treated medically with octreotide. The patient was readmitted once for transaminitis. Peritoneal fluid culture grew *Klebsiella *and *Enterococcus faecium*, which were treated with IV daptomycin, micafungin, and ertapenem with weekly labs. Liver ultrasound and clinic follow-up were weekly, with drain checks every two weeks. Eventually the biliary leak and pancreatic leak resolved with conservative management, and PTC and drain were then removed. Due to R0 resection with clear margin, the patient is being surveilled with regular imaging without adjuvant therapy. Most recently, the patient is doing well, with normalized laboratory values and imaging. He is physically active and has returned to work full-time. Patient immunosuppression was per our center protocol, with no induction therapy, with triple maintenance therapy in the form of tacrolimus, mycophenolate, and steroids that were tapered over six weeks. Infectious prophylaxis, nutritional supplementation, and surveillance labs and imaging were performed per protocol.

## Discussion

BP carries an elevated risk of recurrence and malignant transformation in cases of residual disease after resection of multifocal lesions or diffuse disease involving the intrahepatic and extrahepatic biliary system. Therefore, complete removal of the biliary epithelium with liver transplantation and pancreaticoduodenectomy is considered the only curative treatment in these cases [5]. However, outcomes are not well understood, with prognosis varying widely. They depend on the extent, depth of invasion, and presence of positive lymph nodes. Vibert et al. assessed outcomes in two select cases of diffuse BP without infiltrative invasion or positive lymph nodes. They determined that initial partial resection as a first step to eliminate the presence of advanced tumor invasion and/or positive lymph nodes with subsequent liver transplantation, and/or pancreaticoduodenectomy if indicated, led to successful outcomes and minimal recurrence. The conclusions of this study aimed to improve historically poor preoperative assessments of BP as both endoscopic and percutaneous biopsies have failed to establish the diagnosis of malignancy or the degree of invasion in biliary tumors [5].

We present a rare case of diffuse IPMN-B in the absence of both primary sclerosing cholangitis and prior liver transplantation without a tissue diagnosis of CC prior to surgical intervention. Despite numerous negative biopsies, the potential of malignant transformation into CC was extremely concerning considering that the IPMN-B was diffuse and included the entirety of the biliary epithelium. Due to concern for malignant transformation in the presence of diffuse IPMN-B with both intrahepatic and extrahepatic involvement, OLT and pancreaticoduodenectomy were performed within the same admission according to the original surgical plan. This allowed for complete excision of the diffusely affected biliary tree. The procedures were completed at the same hospital admission, though in a staged fashion, given the complexity and length of each surgery, time under anesthesia, and patient stability. It was not until after OLT, during which the entire resected liver specimen was evaluated, that a small focus of 1-2 mm of CC was identified.

OLT and pancreaticoduodenectomy procedures are incredibly complex, lengthy, and tenuous procedures to perform, each with significant morbidity and mortality. OLT has a short-term mortality of 10-15% at baseline [6]. When completed in addition to a pancreaticoduodenectomy, which carries a mortality rate of 1-5% itself [7], the risks are compounded. Therefore, a staged procedure was selected to monitor early graft function and avoid performing pancreaticoduodenectomy in the setting of coagulopathy and multiple pressors, which is a common condition immediately in the first few hours post-OLT. This staged approach allowed for a period of patient stabilization prior to completion of the pancreaticoduodenectomy procedure.

The patient’s postoperative course was complicated by biliary and pancreatic leaks, a known complication after such procedures that occurs in approximately one quarter of cases [8]. Additionally, our patient was readmitted for transaminitis with a peritoneal fluid culture positive for *Klebsiella* and *E. faecium*. Post- OLT infections are a well-known and common complication occurring in more than 50% of OLT patients, with bacterial infections accounting for around 70% of cases [9]. Our patient has since recovered well, and the small focus of CC that was eventually found was completely resected.

## Conclusions

To our knowledge, this presentation sequence and surgical strategy have not been previously described. This case can provide insights into the topic of managing IPMN-Bs, particularly with diffuse biliary tree involvement. With such a rare disease with widely varied presentations, consensus on a set treatment algorithm is nearly impossible to establish. This case report describes one treatment pathway we found to be successful in our patient’s scenario.
